# High energy level diet improves the growth performance and rumen fermentation of yaks in cold weather

**DOI:** 10.3389/fvets.2023.1212422

**Published:** 2023-07-21

**Authors:** Yanbin Zhu, Guangming Sun, Luosang-dunzhu, Xin Li, Luosang-zhaxi, Suolang-zhaxi, Suolang, Ciyang, Cidan-yangji, Basang-wangdui, Feng Pan, Quanhui Peng

**Affiliations:** ^1^Institute of Animal Science and Veterinary, Tibet Academy of Agriculture and Animal Husbandry Science, Lhasa, China; ^2^Linzhou Animal Husbandry and Veterinary Station, Lhasa, China; ^3^Institute of Animal Nutrition, Key Laboratory of Bovine Low-Carbon Farming and Safety Production, Sichuan Agricultural University, Chengdu, China

**Keywords:** cold weather, dietary energy level, growing yak, methanogenic archaea, metabolomics, rumen fermentation

## Abstract

To date, no research has been done on energy requirements for yaks in Tibetan cold weather. The findings of the current study provide proper energy requirements for yaks would facilitate scientific feeding of fattening yaks in cold weather. The metabolomics and 16s rRNA sequencing technologies were used to explore the underlying mechanism that affects the growth performance of yaks fed with different energy levels of diet in cold weather. Three groups of yaks (141.7 ± 3.34 kg) were fed with diets containing metabolizable energy 7.20, 7.89, and 8.58 MJ/kg DM (dry matter) and named the low-, medium-, and high-energy groups, respectively. The results showed that the average daily feed intake of the high-energy group was higher than that of the low-energy group (*p*  = 0.006). Plasma aspartate aminotransferase (*p*  = 0.004), alanine aminotransferase (*p*  < 0.001), and interferon-γ (*p*  < 0.001) in the high-energy group were lower than in the low-energy group. In contrast, superoxide dismutase (*p*  < 0.001), immunoglobulin G (*p*  < 0.001), and interleukin 2 (*p*  = 0.002) were higher than the low-energy group. The rumen microbial protein (*p*  = 0.025), total volatile fatty acids (*p*  = 0.029), and neutral detergent fiber digestibility (*p*  = 0.050) in the high-energy group were higher than in the low-energy group, whereas the acetate: propionate ratio (*p*  = 0.001) and ammonium nitrogen (*p*  = 0.001) were lower than in the low-energy group. The plasma metabolomics results displayed that yaks fed with a high-energy diet augmented the metabolism of arginine, proline, purine, taste transduction, pyrimidine, and glutathione pathways. The relative abundance of *Methanobrevibacter* in the high-energy group was lower (*p*  < 0.001), whereas the relative abundance of *Methanosphaera* (*p*  < 0.001) was higher than in the low-energy group. The results of the current study suggest that a high-energy diet in growing yaks during the cold season can improve growth performance, rumen microbial protein synthesis, antioxidants, and immunity.

## 1. Introduction

Yaks (*Bos grunniens*) are domesticated on the Qinghai-Tibetan Plateau with a population of 17.6 million, and play an essential role in the life of the local herdsman by providing different types of material such as milk, meat, manure, wool, and hide (1). Most of the yak population are in the Sichuan, Tibet, and Qinghai provinces of China and are domesticated under a traditional management system (2). Over time, the farming system of yaks has changed from an old traditional grazing system to a three-dimension system that comprises a high-altitude zone grazing system, a mid-altitude supplementation of nutrients with grazing, and a low-altitude zone with a fattening indoor feeding system as a result of development and social lifestyle changes among the local population (1). With improved breeding, feeding, farming techniques, and advances in research, the indoor fattening of yaks is surging, and different research trials have been done to study the effects of the different concentrations of the nutritional diet on indoor feeding in yaks (3, 4). In plateau areas, a diet containing high energy levels is more crucial than a high protein diet to support life. Consequently, the studies have chiefly focused on the effects of energy feeding on yaks (3, 5). Different studies have concluded that yaks have the ability to efficiently utilize protein compared to other bovine and caprine animals in cold and harsh environments with low forage availability (6, 7); subsequently, the protein requirement of yaks is lower than that for other animal species.

Bai et al. (8) reported the enteric methane emissions from growing yak calves aged 8–16 months, and suggested that methane emissions in yaks tended to be lower than that of cattle and buffalo calves when fed with similar diets, and that methane emissions in yaks can be predicted by dry matter intake. Comparisons of methane emissions have also been made between grazing and barn-fed yaks, and the results showed that supplementally fed yaks emitted less methane than grazing yaks, and methane emissions decreased as the ratio of concentrate to forage increased (9). Most recently, Liu et al. (10) investigated the effect of different energy levels of diets on methane emissions, trying to explain the methane production differences from a microbial perspective.

A metabolomics approach study was conducted on the Qaidam yellow cattle, dzomo, and yaks by Zhao and Zhao (11), and reported that there were significant differences observed in the concentrations of sugars and the amino, carboxylic, and bile acids among the groups. These results were associated with the microbial composition in the rumen. It is hypothesized that the composition of microorganisms, including bacteria and archaea, in the rumen of yaks changes in the context of variation in dietary energy levels.

Therefore, the current research was conducted to investigate the influence of the different dietary energy levels in the cold season on growth performance, rumen fermentation characteristics, metabolomics, and archaea composition of yaks.

## 2. Materials and methods

### 2.1. Animal ethical statement

All the animals were handled as per the rules and regulations of the animal care guidelines of the Animal Husbandry and Veterinary, Tibet Academy of Agricultural and Animal Husbandry Sciences.

Research trial approval code (TAAAHS-2020-173).

### 2.2. Location of the current trial

The current research trial was conducted at the Linzhou yak demonstration farm, Linzhou, Lhasa City, Tibet, which has an altitude of 3,335 m (29°45′ to 30°08′ N and 90°51′ to 91°28′ E). The current research experiment was conducted from October to December 2021. Linzhou recorded temperatures ranging from −18°C~25°C and 50% humidity during the trial period.

### 2.3. Research design and diet

Twenty-four 2-year-old yaks were separated into three groups as per body weight (BW) in a randomized complete block design. The animals were fed with low energy [7.20 MJ/kg dry matter (DM) LEG], medium energy (7.89 MJ/kg DM: MEG), and high energy (8.58 MJ/kg DM: HEG) diets in the total mixed ration (TMR). The chemical composition and ingredients of the diets are mentioned in Table 1. Before the start of the animal experiment, a 15-day adaptation period was provided to all the experimental animals in the pen (7 m × 5 m). On experiment days, the formulated diet (TMR) was provided twice, at 0900 and 1,700 h, and freshwater was provided to animals *ad libitum* during the experiment period.

**Table 1:** Ingredients and nutritional level of experiment diets.

Item		Treatments^1^	
	LEG	MEG	HEG
Alfalfa hay	20.00	16.66	13.33
Oat hay	20.00	16.66	13.33
Full corn silage	20.00	16.66	13.33
Corn	29.00	38.50	48.00
Rapeseed oil	0.50	0.50	0.50
Wheat bran	5.00	4.50	4.00
Soybean meal	1.50	1.50	1.50
Cottonseed meal	0.50	1.00	1.50
Rapeseed meal	0.50	1.00	1.50
Calcium carbonate	1.65	1.58	1.45
Calcium hydrogen phosphate	0.35	0.44	0.56
Premix^2^	1.00	1.00	1.00
ME, MJ/kg DM^3^	7.20	7.89	8.58
CP, %	9.80	9.85	9.91
EE, %	2.90	3.03	3.16
NDF, %	34.89	30.87	26.84
ADF, %	19.70	17.19	14.67
ADL, %	2.01	1.93	1.84
NFC, %	33.95	37.73	41.99
Starch, %	28.31	33.04	37.77
Ash, %	4.81	4.31	3.82
Ca, %	0.88	0.86	0.83
P, %	0.58	0.57	0.56

1 LEG, low-energy group; MEG, medium-energy group; HEG, high-energy group.

2 The premix provides per kg diet: 10 mg Cu in the form of sulfate, 60 mg Zn in the form of sulfate, 50 mg Mn in the form of sulfate, 50 mg Fe in the form of sulfate, Co in the form of chloride 0.2 mg, I in the form of iodate 0.5 mg, Se in the form of selenite 0.3 mg, and Vitamin A 10,000 IU, Vitamin D_3_ 2,000 IU, and Vitamin E 60 IU.

3 The ME value of TMR was calculated based on the available ME data of the ingredients. CP, EE, NDF, ADF, ADL, Ca, and P were determined values. NFC=DM-(EE% + CP% + CP% + ASH%).

DM, dry matter; ME, metabolizable energy; CP, crude protein; NDF, neutral detergent fiber; NFC, non-fiber carbohydrate; ADF, acid detergent fiber; ADL, acid detergent lignin; Ca, calcium; P, phosphorus.

### 2.4. Growth performance

On day 1 of the experiment, the initial body weight (IBW) of all animals was recorded, and on the last day of the trial, the final body weight (FBW) of animals was recorded. Average daily gain (ADG) was calculated by subtracting the IBW from the FBW and dividing it by 56 days. The growth trial lasted 56 days. The amount of feed provided and leftovers were recorded for all yaks, and the average daily feed intake (ADFI) was calculated based on the DM content.

### 2.5. Apparent nutrient digestibility

Before the start of the digestion trial, a thick plastic sheet was used to cover the floor area of the pen to reduce feces pollution. In the last 4 days of the growth performance trial, plastic buckets were used to collect the feces of the yaks immediately after defecation. Feeds and feces were weighed daily before the 0900 h feed. Subsamples of 3% daily feces output were retained and sulfuric acid 10% was added for nitrogen retention. All the samples were kept at −20°C for later analysis.

According to the procedure of the Association of Official Analytical Chemists (AOAC), the dry matter, crude protein, and acid detergent fiber of the diet and fecal samples were measured as the AOAC methods (DM = 930.15;CP = 984.13;ADF = 935.29) (12). By adopting a heat-stable alpha-amylase process and expressing the results without residual ash, neutral detergent fiber (NDF) and acid detergent fiber (ADF) were examined (13).

### 2.6. Blood collection and analysis

Blood samples (10 mL) of the animals were collected from a jugular vein puncture in a tube containing anticoagulant (ethylenediaminetetraacetic acid) on d 56 before feeding to obtain the plasma. These blood samples were centrifuged at 3,000 × *g* for 15 min at 4°C, and later the plasma was kept at −20°C until further analysis. An automatic bioanalyzer (Hitachi 7200, Hitachi Group, Tokyo, Japan) was used to determine the concentrations of glucose (GLU), triglycerides (TG), albumin, globulin, blood urea nitrogen, total protein, alanine aminotransferase (ALT), aspartate aminotransferase (AST), and alkaline phosphatase in the plasma samples.

Additionally, commercial kits (Jiancheng, Bioengineering Institute of Nanjing, China) were used to determine the antioxidant profile among all the groups per the instructions mentioned. Different Elisa kits from Sigma Chem. Co. were used to analyze the content of interleukin 2, interferon, tumor necrosis factor, and immunoglobulins A, M, and G. O-phenylenediamine was the substrate. An ELISA reader was used to quantify absorbance.

### 2.7. Rumen fermentation characteristics

On the last day of the trial (day 56), ruminal fluid (200 mL) was collected from the animals using a stomach tube at 2, 4, and 6 h post-feeding. The ruminal fluid was separated from the feed particles using four layers of gauze, and a digital pH meter (PHS-3C, Shanghai, China) was used to measure the pH of the ruminal fluid. Afterward, centrifuge machines were used to centrifuge the ruminal fluid at 1,200 × *g* for 15 min. The liquid samples were kept at −20°C until further analysis after adding perchlorate liquid (5%) for the removal of protein. To neutralize the fluid, potassium hydroxide (0.115 mol/L) was used and centrifuged at 400 × *g* for 10 min for the measurement of the ammonia nitrogen (NH_3_-N) and microbial protein (MCP). In contrast, a separate fluid was used to analyze the volatile fatty acid (VFA). The HPLC organic acid analysis system (LC-20A, Shimadzu, Kyoto, Japan) was used to analyze the volatile fatty acid. Ruminal NH_3_-N was measured using the indophenol colorimetric method as designated earlier by Chaney et al. (14); according to the approach described by Makkar et al., microbial protein (MCP) was analyzed by the purine base protocol (15).

### 2.8. Rumen methanogens

#### 2.8.1. Extraction of the DNA

With a DNeasy PowerSoil Kit (Qiagen, Valencia, CA, United States), the total genomic DNA was removed from the rumen fluid in accordance with the manufacturer’s recommendations. DNA was extracted from the elution column with TE buffer, and by 0.8% NanoDrop ND-1000 spectrophotometer (Nyxor Pharmacia, Paris, France), the concentration and quality of the DNA extracted were detected. The ultrapure water 10 ng/μL was used to dilute the extracted all DNA samples and were kept at −20°C for further analysis.

#### 2.8.2. Sequences, amplification of PCR, and generation of Illumina library

Using rumen fluid whole DNA as a template, the V4 variable region of 16S rRNA was amplified, selecting A516F (5′-TGYCAGCC GCCGCGGTAAHACCVGC-3′) and U806R (5′-GGACTACHVGG GTWTCTAAT-3′) as the primer pair for the archaea (16). The identical reaction technique utilized for PCR amplification of the 16S rRNA genes in archaea was employed for the 16S rRNA gene amplification. The total PCR mixture (25 L) contained 0.5 U of KOD-Plus-Neo (Toyobo, Tokyo, Japan), 10 ng template DNA, 1x PCR buffer, 1.5 mM MgCl2, each deoxynucleoside triphosphate at 0.4 M, and each primer at 1.0 M. The PCR amplification process consisted of 30 cycles of denaturation at 94°C for 1 min, annealing at 54°C for 30 s, elongation at 72°C for 30 s, and lastly, extension at 72°C for 5 min. For each sample, three duplicates of the PCR reactions were pooled, and the combined PCR products and 1/6 volume of the 6X loading buffer were loaded onto 2% agarose gel for detection. For further experiments, samples with a bright main strip between 200 and 400 bp were preferred. The electrophoresis band was purified using the OMEGA Gel Extraction Kit (Omega Bio-Tek, United States). The barcoded amplicons that had been gel purified were then pooled and quantified using a Qubit@ 2.0 Fluorometer (Thermo Scientific). Briefly stated, index codes were added after sequencing libraries were created using the TruSeq DNA PCR-Free Sample Prep Kit in accordance with the manufacturer’s instructions. The Qubit@ 2.0 Fluorometer from Thermo Scientific and the Agilent Bioanalyzer 2100 system were used to evaluate the library’s quality. Finally, the pooled amplicons were sequenced using the Hiseq Illumina Sequencing Platform (Rhonin Biosciences Co., Ltd., Chengdu, China) in paired-end mode (2 × 250 bp), based on the guidelines provided by Caporaso et al. (17).

#### 2.8.3. Bioinformatics analysis

The sequences were examined utilizing the Usearch and QIIME pipelines.^1^ Using FLASH, paired-end readings from the original DNA fragments were combined (18). Sequences were allocated to each sample based on the distinct barcode. The initial phase involved utilizing Trimmomatic and Usearch to filter out low-quality reads (length 200 bp, more than two ambiguous base ‘N’s, or an average base quality score of 30) and shorter sequences where quality scores declined (score 11). All singletons were eliminated after duplicate sequences were discovered since they might be poor amplicons,^2^ which resulted in an overestimated diversity. Using UPARSE algorithms, sequences were grouped into operational taxonomic units (OTUs) at a 97% identity criterion (19), and, utilizing the Uchime method, exemplary sequences were selected and possible chimeras eliminated (20). Taxonomies were assigned using the Silva database and the uclust classifier in QIIME. Using PyNAST, which is incorporated in QIIME, representative sequences were aligned. Good’s coverage and rarefaction curves were determined to calculate the coverage and sampling effort. Mothur was utilized to determine the diversity, evenness, and richness of the archaea population (Simpson index, Shannon-Wiener index, PD, and Chao1). Principal coordinates analysis (PCoA) and cluster heatmap were performed to evaluate significant changes between samples.

### 2.9. Blood plasma metabolomics

Plasma samples were subjected to GC/MS analysis using an Agilent 7890 gas chromatograph system in conjunction with a Pegasus HT time-of-flight mass spectrometer (LECO, St. Joseph, MI), and the protocol was followed as mentioned in the previous study (21).

### 2.10. Statistical analyses

All data for all parameters were collected and summarized in Microsoft Excel 2010. Growth performance, plasma parameters, rumen fermentation characteristics, and nutrient digestibility results were analyzed using SAS 9.3 according to a randomized complete block design (SAS Inst. Inc., Cary, NC, United States, 2012). The mixed model included the fixed effect of dietary energy level and block as a random effect:


Yij=μ+Ti+Bj+eij.

where: *Yij* = dependent variable; *μ* = overall mean; *Ti* = fixed effect of energy level within block; *Bj* = random effect of block j; *eij* = random residual variation.

The least-squares means and mean standard deviations were used to show the data. The *p* < 0.05 was used to define significance, while *p* < 0.05 to ≤0.10 was used to define a trend. LECO Corporation’s Chroma TOF 4.3X software and the LECO-Fiehn Rtx5 database were used to analyze the metabolomics data for raw peak extraction, data baseline filtering and calibration, peak alignment, deconvolution analysis, peak identification, and peak area integration. SIMCA-P software (V 14.0, Umetrics, Umea, Sweden) was used to conduct a multivariate analysis of the orthogonal correction partial least squares discriminant analysis (OPLS-DA). Differentially expressed metabolites between low- and high-energy group treatments were identified based on variable importance in projection (VIP) from OPLS-DA analysis and Kyoto Encyclopedia of Genes and Genomes (KEGG, http://www.genome.jp/kegg/, VIP > 1 and *p* < 0.05) analysis was conducted to view the enriched pathways of different metabolites.

## 3. Results

### 3.1. Growth performance

The growth performance results displayed a significant difference in ADFI among all the groups (*p* = 0.006), and the ADFI of high energy group was greater compared to low-energy group (*p* < 0.05); no difference was observed between low- and medium-energy groups or the medium- and high-energy groups (*p* > 0.05). However, in FBW, a significantly increased tendency was observed among all groups (*p* = 0.069), and the highest was observed in the high-energy group. In the ADG (*p* = 0.201) and feed conversion ratio (FCR) (*p* > 0.05) results among all three groups, no significant differences were observed (Table 2).

**Table 2:** Effects of different dietary levels of energy on the growth performance of growing yaks.

Item	Treatments			*P*-value
	LEG	MEG	HEG	
IBW, kg	139.08 ± 2.311	140.19 ± 4.211	145.96 ± 3.526	0.346
FBW, kg	164.54 ± 2.867	168.08 ± 4.986	179.04 ± 4.862	0.069
ADG, kg	0.45 ± 0.0449	0.50 ± 0.0396	0.59 ± 0.071	0.201
ADFI, kg/d	5.34 ± 0.072^b^	5.52 ± 0.072^ab^	5.69 ± 0.066^a^	0.006
FCR	13.09 ± 1.341	11.90 ± 0.912	11.64 ± 1.577	0.705

IBW, initial body weight; FBW, final body weight; ADG, average daily gain; ADFI, average daily feed intake; FCR, feed conversion ratio. Different lowercase superscript indicates significant difference of *p* < 0.05.

### 3.2. Nutrients apparent digestibility

A significant difference was observed in DM (*p* = 0.006), OM (*p* = 0.019), and NDF (*p* = 0.050) the digestibility of DM, OM and NDF was higher than that of the low-energy group (*p* < 0.05). There was a significant tendency for ADF digestibility among the three groups (*p* = 0.089); the highest was noted in the high-energy group. No significant change was observed in CP digestibility among the three groups (*p* = 0.318; Table 3).

**Table 3:** Effects of different dietary levels of energy on nutrients apparent digestibility of growing yaks.

Item	Treatments			*P-*value
	LEG	MEG	HEG	
DM, %	63.27 ± 0.462^b^	64.47 ± 0.494^ab^	66.11 ± 0.689^a^	0.006
OM, %	65.62 ± 0.546^b^	66.46 ± 0.478^ab^	68.03 ± 0.640^a^	0.019
CP, %	62.38 ± 0.569	62.63 ± 0.537	63.53 ± 0.549	0.318
NDF, %	61.48 ± 0.730^b^	62.30 ± 0.618^ab^	63.35 ± 0.246^a^	0.050
ADF, %	38.10 ± 0.412	38.31 ± 0.498	39.87 ± 0.624	0.089

DM, dry matter; OM, organic matter, CP, crude protein, NDF, neutral detergent fiber, ADF, acid detergent fiber. Different lowercase superscript indicates significant difference of *p* < 0.05.

### 3.3. Blood plasma variables

The results of the GLU (*p* < 0.001), ALT (*p* = 0.004), and AST (*p* < 0.001) found significant differences among all the three groups. The concentration of the GLU in the high-energy group was observed as higher compared with other groups (*p* < 0.05). In contrast, the content of ALT and AST in the low-energy group was greater compared with the medium- and high-energy groups (*p* < 0.05). There was a significant trend in the ALP (*p* = 0.057) and TG (*p* = 0.063) among the three groups (Table 4), and the lowest of TG and ALP was observed in the high-energy group.

**Table 4:** Effects of different dietary levels of energy on the plasma chemical parameters of growing yaks.

Item	Treatments			*P-*value
	LEG	MEG	HEG	
TP, g/L	59.26 ± 1.260	59.88 ± 0.923	60.12 ± 0.546	0.979
ALB, g/L	27.50 ± 0.658	28.89 ± 0.706	29.37 ± 0.277	0.382
GLOB, g/L	31.77 ± 0.870	30.94 ± 0.690	30.75 ± 0.381	0.328
GLU, mmol/L	3.98 ± 0.036^b^	4.11 ± 0.090^b^	4.46 ± 0.084^a^	<0.001
TG, nmol/L	0.10 ± 0.004	0.09 ± 0.005	0.08 ± 0.010	0.063
BUN, mmol/L	3.17 ± 0.067	3.15 ± 0.098	3.05 ± 0.317	0.439
ALT, U/L	3.47 ± 0.268^a^	3.22 ± 0.076^b^	3.15 ± 0.073^b^	0.004
AST, U/L	44.16 ± 1.38^a^	42.17 ± 0.816^b^	40.43 ± 0.640^b^	<0.001
ALP, U/L	43.67 ± 3.400	42.12 ± 2.158	41.12 ± 0.746	0.057

TP, total protein; ALB, albumin; GLOB, globulin; GLU, glucose; TG, triglyceride; BUN, urea nitrogen; ALT, alanine aminotransferase; AST, aspartate aminotransferase; ALP, alkaline phosphatase. Different lowercase superscript indicates significant difference of *p* < 0.05.

### 3.4. Blood plasma antioxidant variables

Compared with the low-energy group, the superoxide dismutase (SOD) in the high-energy group increased by 15.83% (*p* < 0.001). No significant change was observed in total antioxidant capacity (T-AOC) (*p* = 0.330), glutathione peroxidase (GSH-Px) (*p* = 0.129), and malondialdehyde (MDA) (*p* = 0.221) among the three groups (Table 5).

**Table 5:** Effects of different dietary levels of energy on blood plasma antioxidant indices of growing yaks.

Item	Treatments			*P-*value
	LEG	MEG	HEG	
T-AOC, U/mL	11.21 ± 0.337	11.53 ± 0.425	11.93 ± 0.203	0.330
SOD, U/mL	130.51 ± 2.754^b^	145.45 ± 3.410^ab^	151.17 ± 0.398^a^	<0.001
GSH-Px, U/mL	189.99 ± 11.023	206.61 ± 11.363	217.38 ± 14.712	0.129
MDA, nmol/mL	5.34 ± 0.246	5.19 ± 0.184	4.93 ± 0.069	0.221

T-AOC, total antioxidant capacity; SOD, superoxide dismutase; GSH-Px, glutathione peroxidase; MDA, malondialdehyde. Different lowercase superscript indicates significant difference of *p* < 0.05.

### 3.5. Blood plasma cytokines and immunoglobulins variables

The IL-2 content in the high-energy group was higher than in the low- and medium-energy groups (*p* = 0.002). On the contrary, the TNF-α content in the high-energy group was lower than that of the medium- and low-energy groups (*p* < 0.001). The IgG in the high-energy group was higher than in the low- and medium-energy groups (*p* < 0.05), the IgG of medium-energy group was higher than the low-energy group (*p* < 0.05). However, no significant variation was observed in IL-4 (*p* = 0.644), IL-6 (*p* = 0.798), IL-10 (*p* = 0.224), IFN-γ(*p* = 0.221), IgA (*p* = 0.242), and IgM (*p* = 0.545) among all groups (Table 6).

**Table 6:** Effects of different dietary levels of energy on blood plasma cytokines and immunoglobulin of growing yaks.

Item	Treatments			*P*-value
	LEG	MEG	HEG	
IL-2, ng/L	58.13 ± 4.591^b^	61.34 ± 3.656^b^	77.28 ± 1.301^a^	0.002
IL-4, ng/L	73.16 ± 1.580	72.41 ± 2.240	71.04 ± 0.443	0.644
IL-6, ng/L	90.14 ± 3.408	91.98 ± 2.996	92.61 ± 1.066	0.798
IL-10, ng/L	90.14 ± 4.302	91.98 ± 5.830	92.62 ± 1.649	0.224
IFN-γ, ng/L	110.55 ± 4.174	109.64 ± 5.456	119.18 ± 2.001	0.221
TNF-α, ng/L	87.82 ± 1.572^a^	83.01 ± 1.824^ab^	76.20 ± 1.900^b^	<0.001
IgA, g/L	0.64 ± 0.012	0.67 ± 0.020	0.68 ± 0.010	0.242
IgG, g/L	0.20 ± 0.011^c^	0.23 ± 0.006^b^	0.25 ± 0.004^a^	<0.001
IgM, g/L	0.07 ± 0.001	0.07 ± 0.001	0.08 ± 0.001	0.545

IL-2, interleukin 2; IL-4, interleukin 4; IL-6, interleukin 6; IL-10, interleukin 10; IFN-γ, interferon-γ; TNF-α, tumor necrosis factor α; IgA, immunoglobulin A; IgG, immunoglobulin G; IgM, immunoglobulin M. Different lowercase superscript indicates significant difference of *P* < 0.05.

### 3.6. Rumen fermentation characteristics

The dietary energy level had a significant effect on MCP (*p* = 0.025), NH_3_-N (*p* = 0.001), total volatile fatty acids (TVFA) (*p* = 0.029), acetate (*p* = 0.012), butyrate (*p* = 0.030), and acetate: propionate ratio (*p* = 0.010). The NH_3_-N, acetate, and acetate: propionate ratio in the high-energy group was lower than in the low- and medium-energy groups (*p* < 0.05). The TVFA and MCP in the high-energy group were higher than the low- and medium-energy groups (*p* < 0.05). The butyrate content in the medium-energy group was higher than in the low-energy group (*p* < 0.05). In addition, there was a significant trend for propionate (*p* = 0.092) among the three groups to increase, and the highest was observed in the high-energy group (Table 7).

**Table 7:** Effects of different dietary levels of energy on the rumen fermentation characteristics of growing yaks.

Item	Treatments			*P*-value
	LEG	MEG	HEG	
pH	6.71 ± 0.048	6.62 ± 0.050	6.55 ± 0.059	0.122
MCP, g/dL	52.05 ± 1.741^b^	54.75 ± 1.224^ab^	57.12 ± 1.646^a^	0.025
NH_3_-N, mg/dL	4.92 ± 0.163^a^	4.35 ± 0.118^ab^	4.02 ± 0.161^b^	0.001
TVFA, mmol/L	63.27 ± 0.491^b^	65.18 ± 0.543^b^	68.47 ± 0.581^a^	0.029
Acetate, mmol/L	42.80 ± 0.376^a^	42.92 ± 0.433^ab^	44.29 ± 0.763^b^	0.012
Propionate, mmol/L	13.11 ± 0.343	14.25 ± 0.278	17.07 ± 0.338	0.092
Butyrate, mmol/L	5.44 ± 0.112^b^	6.22 ± 0.248^a^	6.33 ± 0.182^ab^	0.030
Acetate: propionate	3.28 ± 0.074^a^	3.02 ± 0.046^b^	2.95 ± 0.089^b^	0.010

MCP, microbial crude protein; NH_3_-N, ammonium nitrogen; TVFA, total volatile fatty acids. Different lowercase superscript indicates significant difference of *P* < 0.05.

### 3.7. Rumen methanogens

**Figure 1:**
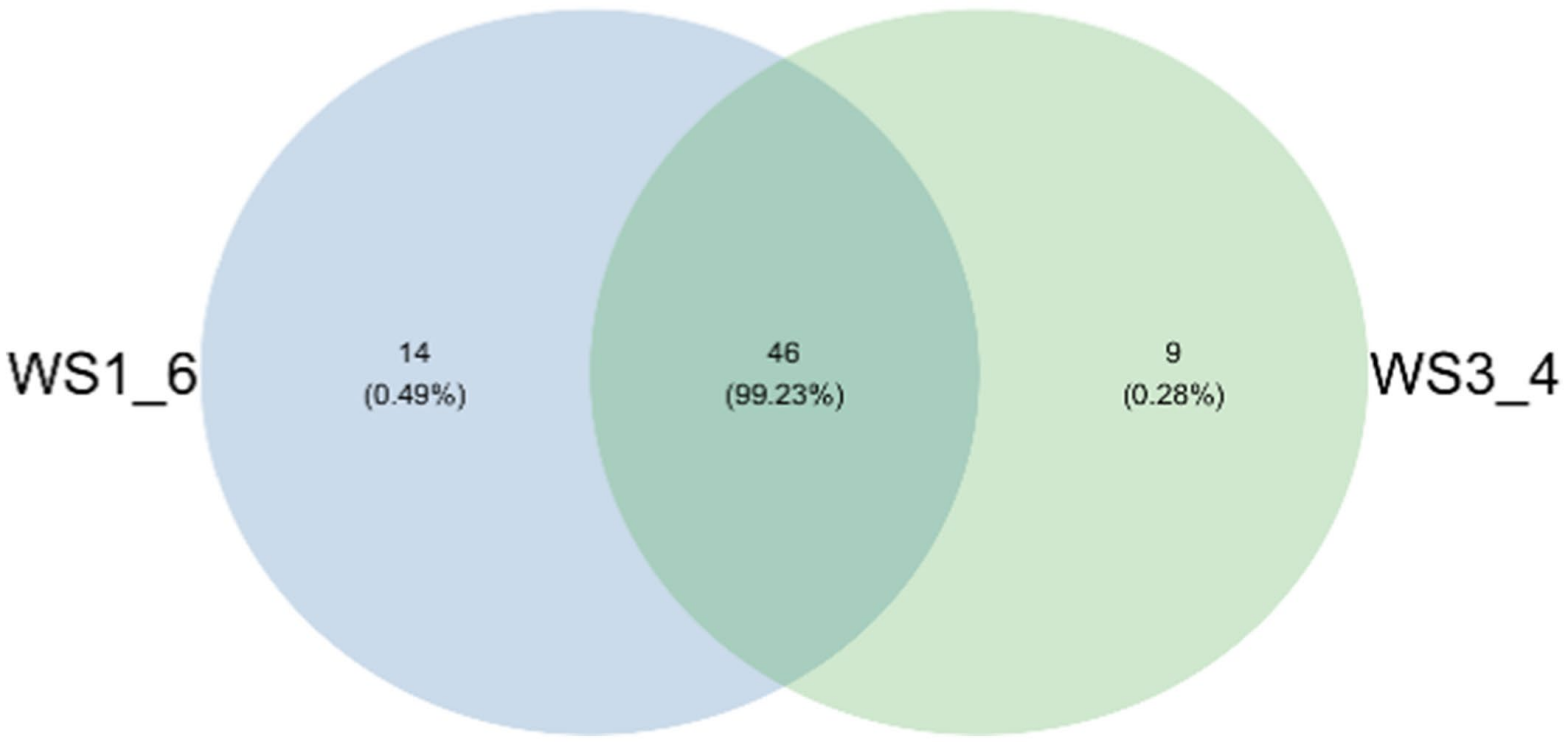
Venn diagram representation of the shared and exclusive methanogenic bacteria OTUs at 97% similarity level between low- (WS1_6) and high- (WS3_4) energy group yaks.

**Figure 2:**
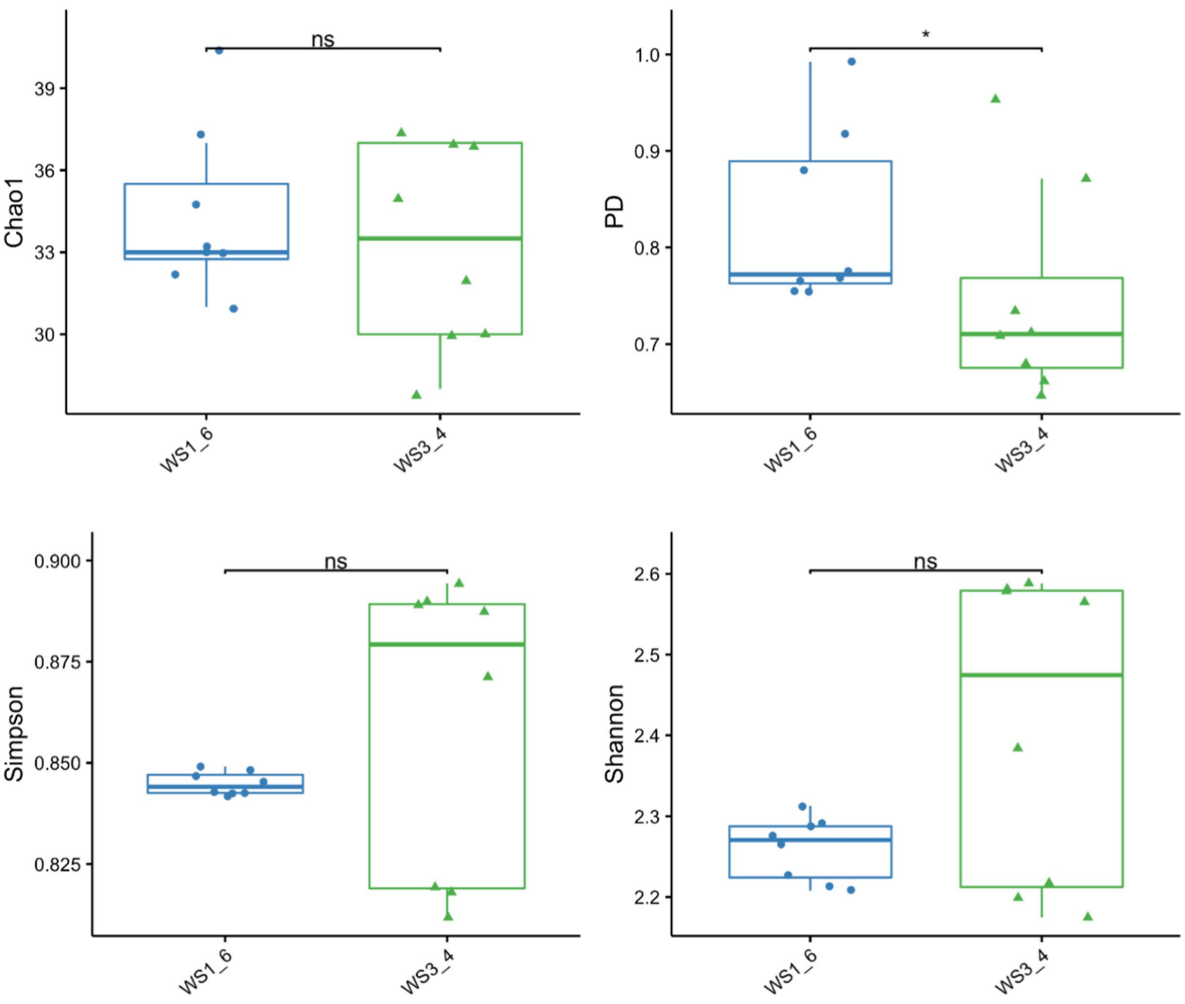
The richness and diversity of methanogenic bacteria estimated using the Chao1, PD, Shannon, and Simpson indices of low- (WS1_6) and high- (WS3_4) energy group yaks.

**Figure 3:**
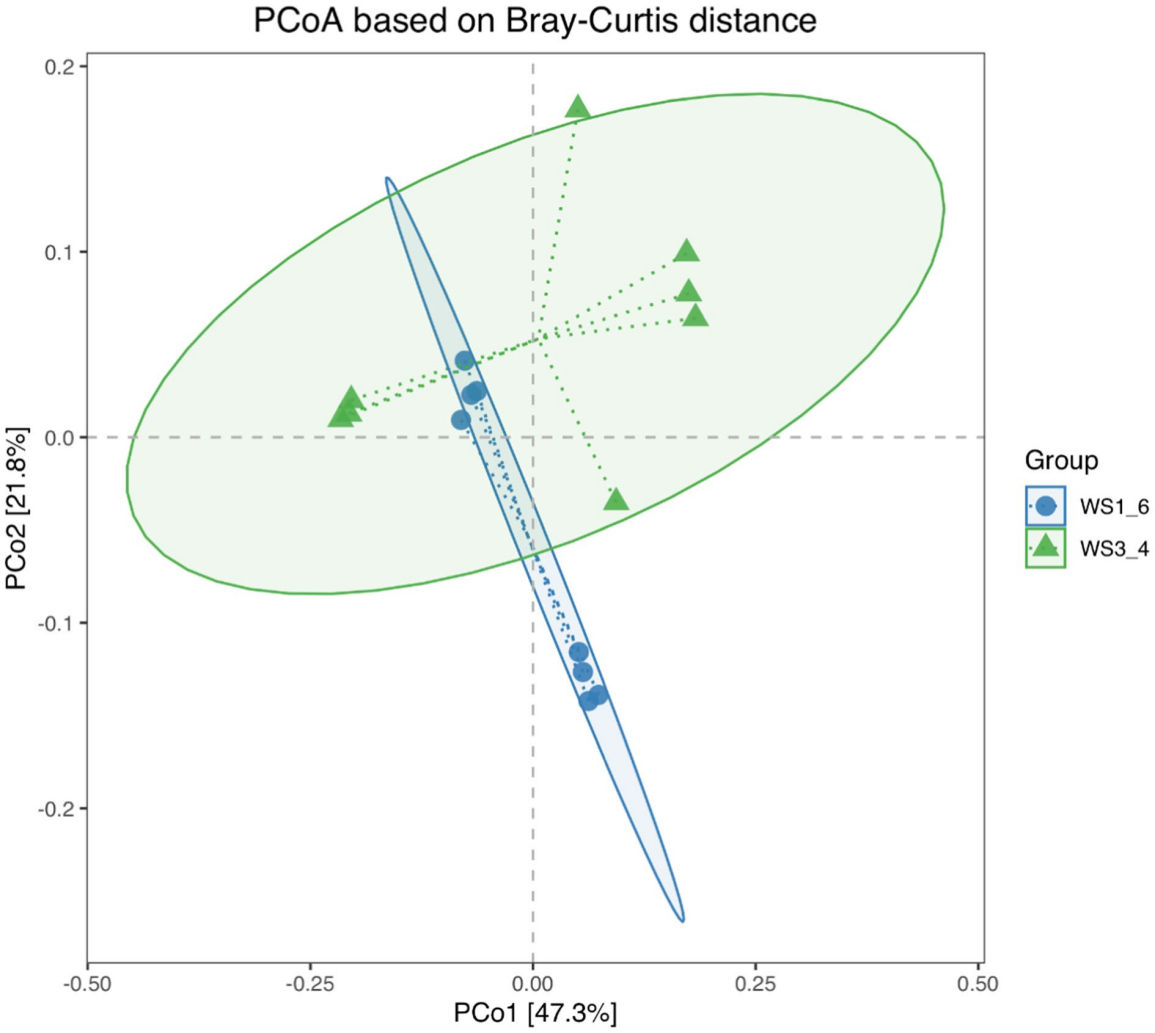
Principal coordinates analysis (PCoA) scores plot of methanogenic bacteria generated using a Bray-Curtis distance analysis of low- (WS1_6) and high- (WS3_4) energy group yaks.

**Figure 4:**
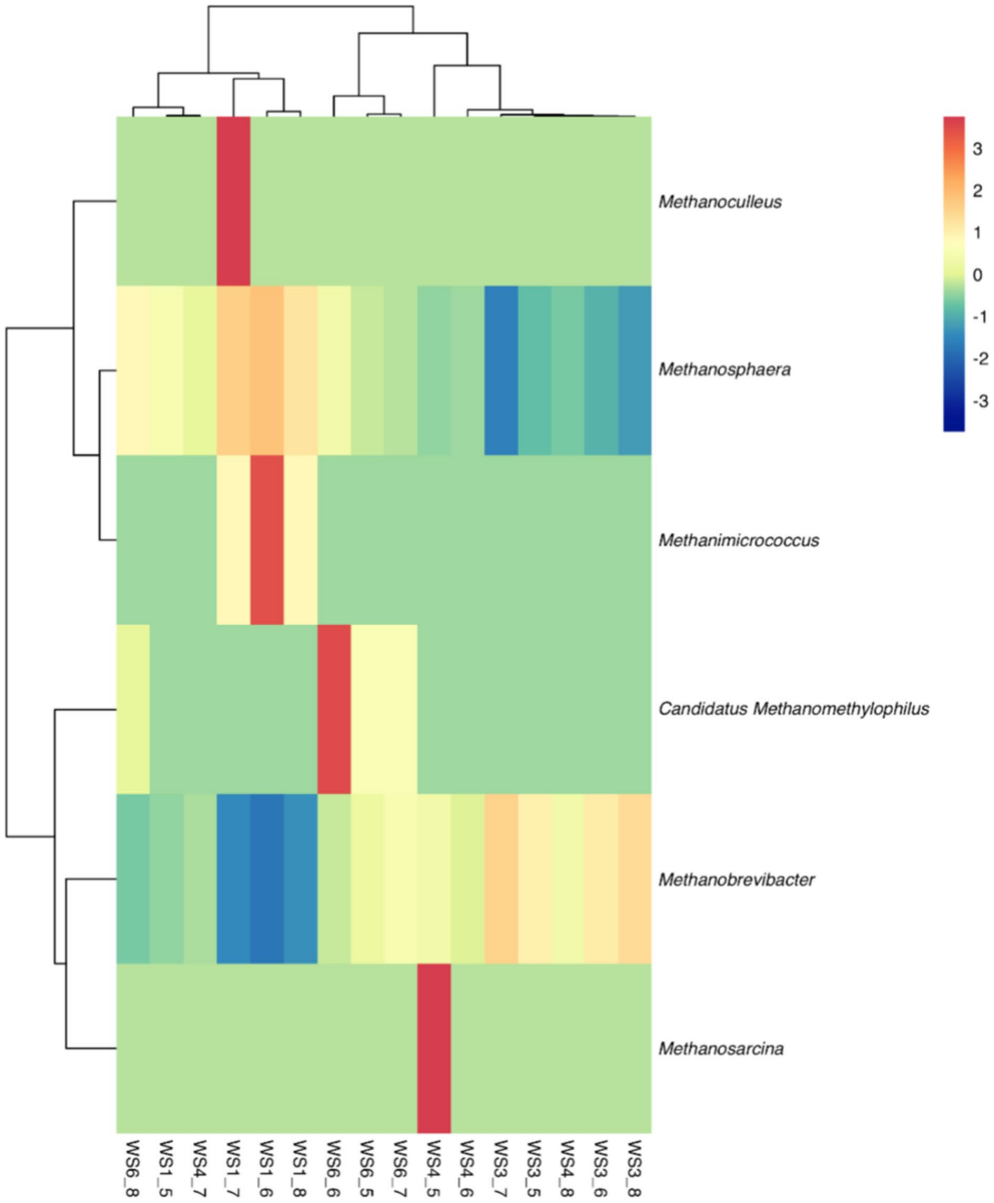
Hierarchical clustering analysis (HCA) and heatmap of the methanogenic bacteria in low- (WS1_6) and high- (WS1_6) energy group yaks. Rows represent samples, and columns represent methanogenic bacteria. Cells were colored based on the relative abundance of methanogenic bacteria; red represents high relative abundance while blue represents low relative abundance and white cells show the intermediate level.

Because most physiological and biochemical indicators in the high-energy group were superior to the low-energy group, these two groups were selected for sequencing. The sequencing coverage and OTU classification results are shown in Supplementary Figure S1 and Figure 1. In total, 46 OTUs were common to the high- and low-energy group, and 14 and 9 OTUs were unique to the high- and low-energy groups, respectively. The Shannon, Simpson, Chao1, and PD indexes were used to estimate the species richness and diversity of methanogenic bacteria. No significant difference was detected in the Chao1, Shannon, and Simpson indices; however, the PD index of the high-energy group was higher compared with that of low-energy group (Figure 2). The PC1 and PC2 explained 47.3 and 21.8% of the total variance, correspondingly (Figure 3). However, the PCoA findings displayed that neither the high- nor low-energy groups clustered together well. The relative abundance of *Methanobrevibacter* in the high-energy group was greater than that of the low-energy group (*p* < 0.001). In contrast, the relative abundance of *Methanosphaera* in the high-energy group was lower than that of the low-energy group (*p* < 0.001; Table 8; Figure 4).

**Table 8:** Effects of dietary energy level on the methanogenic bacteria abundance of growing yaks.

Item	Treatment		*P*-value
	LEG	HEG	
Phylum level			
Euryarchaeota	0.999 ± 0.354	0.997 ± 0.406	0.270
Thaumarchaeota	0.000 ± 0.000	0.002 ± 0.000	<0.001
Genus level			
*Methanobrevibacter*	0.947 ± 0.609	0.966 ± 0.378	0.005
*Methanosphaera*	0.05 ± 1.077	0.03 ± 0.463	<0.001

## 4. Discussion

### 4.1. Feed intake and growth performance

**Figure 5:**
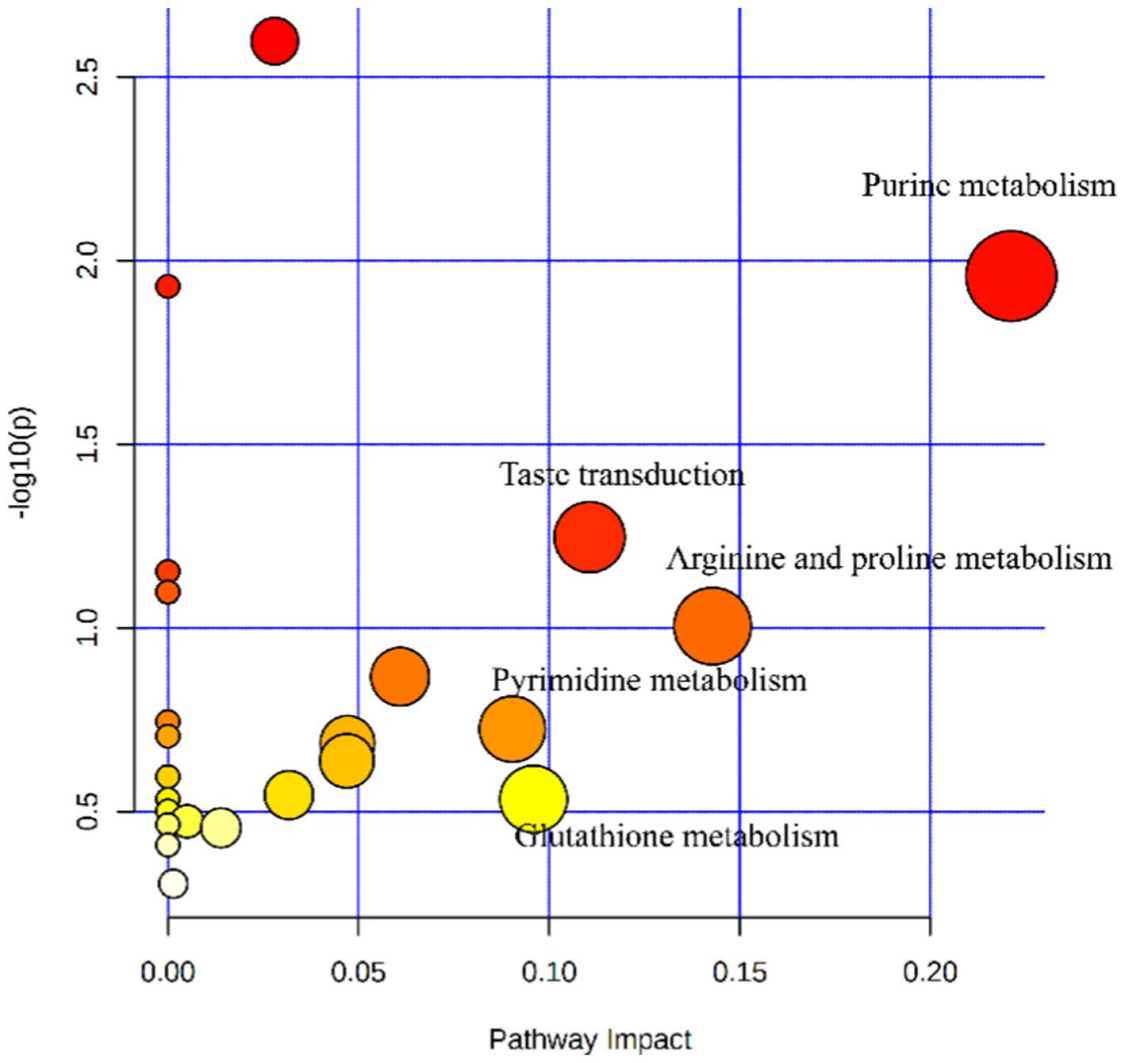
Metabolome map of serum metabolites from yaks (*n* = 8) fed with a high-energy diet vs. a low-energy diet. The X-axis represents the pathway impact and the Y-axis represents the *p*-value. The larger the size of the circle indicates more metabolites enriched in that pathway and the larger abscissa indicates higher pathway impact values. A darker color indicates smaller *p*-values.

Feed efficiency and growth performance are key traits for the yak husbandry and the economy of the country. The growth performance and feed intake of the yaks are influenced by various factors such as housing, environment, diet, and genetic makeup of the animals (22). The results of the current research showed that higher energy levels in the diet of the yaks in the cold season increased the growth performance of the yaks. These results are in line with the earlier study by Liu et al. (5). A recently published paper studied the effect of dietary energy levels on slaughter performance and meat quality and tried to elaborate the mechanism of how dietary energy improves meat quality (3). Typically, as the dietary energy concentration increases, the dry matter intake (DMI) will decrease, which has been reported before by Yang et al. (3). However, the DMI increased as the dietary energy concentration increased in the present study. This may be related to an increase in the digestibility of the diet. Because the dietary energy level was elevated by increasing the amount of corn in the formulation, while the proportion of roughage was decreased, therefore the DM, OM, and NDF digestibility increased, which was consistent with previous reports (5). It has also been proven that less fiber intake results in higher DMI and nutrient digestibility (13), and the plasma metabolomics results showed that the taste transduction pathway of the high-energy diet-fed yaks was enhanced (Table 9; Supplementary Table S1; Figure 5), which was helpful in elevating DMI.

**Table 9:** The detailed results of metabolic pathways and different metabolites for the high-energy diet vs. low-energy diet groups.

Metabolic pathways	Metabolites
Purine metabolism (9)	Xanthine, Hypoxanthine, Guanosine, Guanine, ADP, Adenosine, Adenine, 2′-Deoxyadenosine, (R)(-)-Allantoin
Arginine and proline metabolism (9)	Spermine, Spermidine, N-Carbamoylputrescine, L-Glutamic acid, Glutamic Acid, gamma-Glutamyl-gamma-aminobutyraldehyde, gamma-Aminobutyric Acid, D-Proline, Creatinine
Taste transduction (7)	Saccharin, Norepinephrine, L-Glutamic acid, Glutamic Acid, gamma-Aminobutyric Acid, D-Serine, ADP
Pyrimidine metabolism (5)	Uracil, Thymine, Cytidine, 1-beta-delta-Ribofuranosyl-Cytosine, Beta-Alanine
Glutathione metabolism (4)	Spermine, Spermidine, L-Glutamic acid, Glutamic acid

### 4.2. Blood plasma variables

Blood parameters reflect normal health and metabolic functions in animals. Previous reports have shown that if energy intake is insufficient, it will lead to a decrease in blood glucose (23), and decreased blood glucose was observed in starved yaks in our previous study (24). With increased starch or NFC intake, an increase in rumen propionate or acetate: propionate ratio led to a rise in blood glucose concentration. However, the variation of blood glucose concentration was in the normal range, 3.9~5.3 mmol/L (25).

The content of ALT, AST, and ALP are measures of liver health, and if they are elevated, it means that the liver is in a state of stress (26). This study observed that the three enzymes decreased with the increase in dietary energy, which meant the stress on yak’s liver was less. In addition, the elevated plasma SOD concentration meant that the antioxidant capacity of the yaks was enhanced (27). An increase in plasma IL-2 and a decrease in TNF-α and IFN-γ in the high-energy group meant that the pro-inflammatory response was inhibited, while the anti-inflammatory response was enhanced, and the yak immunity function was improved. The results of metabolomics also showed that the high-energy diet enhanced arginine and proline metabolism and glutathione metabolism pathways, which meant that the yak antioxidant and immune functions were enhanced (28, 29) (Table 9; Supplementary Table S1; Figure 5). We speculated that under the conditions of this experiment, yaks in the low-energy group ingested low levels of nutrients, which were insufficient to maintain body health, and increasing the nutrient intake level improved the body’s antioxidant capacity and immunity.

### 4.3. Rumen fermentation parameters

The parameters of rumen fermentation showed the status of nutrient digestion and metabolism and even the utilization efficiency of energy and protein to a certain extent. The variation trend of TVFA, acetate, propionate, and acetate: propionate ratio with dietary energy concentrations in our study were almost exactly the same as previously reported (5). The highest TVFA and propionate and lowest acetate and acetate: propionate ratio were observed in the high energy group. From the perspective of diet structure, our means of increasing the dietary energy concentration was mainly by increasing the proportion of corn and reducing the proportion of forage. Increasing starch or NFC will increase propionate production, reducing the acetate: propionate ratio. We observed that NH_3_-N decreased with dietary energy concentration, whereas (5) reported that NH_3_-N increased with the increase of energy concentration, while the MCP change trend was consistent with our results. This may be due to the different compositions of the two diets and the different degradation rates of dietary proteins. Our metabolomics results indicated that the purine and pyrimidine metabolic pathways were promoted with the increase of dietary energy concentrations, which coincided with the increased MCP variation trend (Table 9; Supplementary Table S1; Figure 5).

### 4.4. Rumen methanogens

It has been stated that *Methanobrevibacter* is the utmost abundant hydrogenotrophic methanogenic archaea in the rumen of yaks (30, 31), and the relative abundance of *Methanobrevibacter* augments as the age of yaks increases (32). Our study observed that as the dietary energy concentration increased, the relative abundance of *Methanobrevibacter* increased though the relative abundance of *Methanosphaera* declined. This implied that nutritional levels could regulate the rumen archaeal composition. This was also confirmed by Pang et al. (33), who reported that the *Methanobrevibacter* content was higher in the dietary forage-to-concentrate ratio 65:35 group than in the 80:20 group. It was reported that at the species level, *Methanobrevibacter gottschalkii* was relatively abundant in the rumen of cows with high methane production. At the same time, *Methanobrevibacter ruminantium* was relatively abundant in the rumen of cows with low methane production (34). Therefore, the relative abundance of *Methanobrevibacter* increased with dietary energy concentration, and it is still possible to lead to a decrease in methane production, which warrants further study.

## 5. Conclusion

We have concluded from the findings of the current trial that higher energy levels in the diet of the Yaks in the cold season increase glucose metabolism and improve the antioxidant and immunity profile and growth performance. The energy level ME 8.58 MJ/kg DM in the cold season is recommended for the healthy growth and performance of yaks in the cold season.

## Data availability statement

The datasets presented in this study can be found in online repositories. The names of the repository/repositories and accession number(s) can be found in the article/Supplementary material.

## Ethics statement

The experimental protocol used in the present study was approved by the Animal Policy and Welfare Committee of the Agricultural Research Organization of Tibet Autonomous Region, China, and was in accordance with the guidelines of the Animal Care and Ethical Committee of the Institute of Animal Husbandry and Veterinary Medicine, Tibet Academy of Agriculture and Animal Husbandry Science (TAAAHS-2020-173).

## Author contributions

YZ: writing-original draft and writing-review. YZ and QP: formal analysis. YZ, GS, L-d, XL, L-z, LS, Ciyang, and C-y: investigation. B-w and QP: conceptualization and supervision. LS: project administration. QP: editing. B-w: validation and funding acquisition. All authors contributed to the article and approved the submitted version.

## Funding

The current research was funded by the Yak Breeding Innovation and Healthy Farming, funding no XZ202101ZD0002N, the Kelsang Tang Yak Breeding and Efficient Propagation in Linzhou County (QYXTZX-LS2020-01), the National Beef Cattle Yak Industry Technology System (CARS-37), and the Natural Science Foundation of Sichuan Province (2022NSFSC0064).

## Conflict of interest

The authors declare that the research was conducted in the absence of any commercial or financial relationships that could be construed as a potential conflict of interest.

## Publisher’s note

All claims expressed in this article are solely those of the authors and do not necessarily represent those of their affiliated organizations, or those of the publisher, the editors and the reviewers. Any product that may be evaluated in this article, or claim that may be made by its manufacturer, is not guaranteed or endorsed by the publisher.

## Footnotes

^1^ http://drive5.com/uparse/

^2^ http://www.drive5.com/usearch/manual/singletons.html
