# Supplementary material for: High energy level diet improves the growth performance and rumen fermentation of yaks in cold weather
Source: Front Vet Sci. 2023 Jul 21;10:1212422. doi: 10.3389/fvets.2023.1212422 (PMC10402921; doi:10.3389/fvets.2023.1212422)
Supplement: Supplementary file 1 [file Data_Sheet_1.docx]

***Supplementary Material***

**Supplementary Table 1**. Significantly different metabolites that enriched in the pathways of yaks fed with high energy diet (S1_6) versus yaks fed with low energy diet (S3_4).

| Name | Formula | Calc. MW | RT [min] | VIP | FC S1_6/S3_4 | P value |
| --- | --- | --- | --- | --- | --- | --- |
| UNII:X8SB352YP5 | C12 H18 N6 | 246 | 19.98 | 2.19 | 4.08 | 0.019 |
| gamma-Glutamyl-gamma-aminobutyraldehyde | C9 H16 N2 O4 | 216 | 0.98 | 1.60 | 3.01 | 0.034 |
| US4204000 | C6 H6 N2 S | 138 | 6.79 | 1.51 | 2.61 | 0.000 |
| Norepinephrine | C8 H11 N O3 | 169 | 4.91 | 1.47 | 2.28 | 0.020 |
| Adenosine | C10 H13 N5 O4 | 267 | 1.42 | 1.61 | 2.16 | 0.046 |
| quindecamina | C30 H38 N4 | 454 | 9.30 | 3.41 | 2.13 | 0.012 |
| Hypoxanthine | C5 H4 N4 O | 136 | 1.21 | 1.12 | 1.93 | 0.026 |
| D-Proline | C5 H9 N O2 | 115 | 1.70 | 1.34 | 1.92 | 0.018 |
| Guanine | C5 H5 N5 O | 151 | 1.71 | 1.29 | 1.91 | 0.024 |
| Glutamic Acid | C5 H9 N O4 | 147 | 1.16 | 1.53 | 1.90 | 0.045 |
| 2'-Deoxyadenosine | C10 H13 N5 O3 | 251 | 1.53 | 1.71 | 1.90 | 0.015 |
| Creatinine | C4 H7 N3 O | 113 | 0.85 | 3.48 | 1.87 | 0.023 |
| C10E8 | C26 H54 O9 | 510 | 10.48 | 2.46 | 1.84 | 0.015 |
| ADP | C10 H15 N5 O9 P2 S | 443 | 1.82 | 1.64 | 1.82 | 0.019 |
| 2-Methoxy[1,3]thiazino[6,5-b]indol-4(9H)-one | C11 H8 N2 O2 S | 232 | 8.80 | 3.12 | 1.81 | 0.023 |
| Uracil | C4 H4 N2 O2 | 112 | 1.18 | 1.54 | 1.81 | 0.032 |
| Xanthine | C5 H4 N4 O2 | 152 | 1.31 | 1.45 | 1.79 | 0.007 |
| Propyphenazone | C14 H18 N2 O | 230 | 7.40 | 1.67 | 1.79 | 0.005 |
| Guanosine | C10 H13 N5 O5 | 283 | 1.71 | 1.29 | 1.76 | 0.040 |
| Spermine | C10 H26 N4 | 202 | 0.77 | 1.43 | 1.74 | 0.016 |
| (R)(-)-Allantoin | C4 H6 N4 O3 | 158 | 0.98 | 1.60 | 1.67 | 0.008 |
| 3,6,9,12,15,18-Hexaoxaoctacosan-1-ol | C22 H46 O7 | 422 | 10.50 | 3.25 | 1.65 | 0.026 |
| 2-{2-[2-(Decyloxy)ethoxy]ethoxy}ethanol | C16 H34 O4 | 290 | 10.46 | 3.58 | 1.56 | 0.018 |
| N,N-Bis(2-hydroxyethyl)dodecanamide | C16 H33 N O3 | 287 | 9.05 | 2.99 | 1.55 | 0.048 |
| Bacillamidin G | C17 H35 N O | 269 | 14.12 | 3.60 | 1.52 | 0.027 |
| D-Serine | C3 H7 N O3 | 105 | 0.90 | 1.90 | 1.11 | 0.037 |
| UNII:TYL476W27Y | C18 H30 O | 262 | 12.07 | 2.48 | 0.62 | 0.047 |
| 3-Hydroxycyclohexanone | C6 H10 O2 | 114 | 5.02 | 1.13 | 0.62 | 0.021 |
| Linoleamide | C18 H33 N O | 279 | 12.07 | 2.42 | 0.60 | 0.048 |
| 3-Vinyl-7-oxabicyclo[4.1.0]heptane | C8 H12 O | 124 | 6.41 | 1.48 | 0.60 | 0.042 |
| Bis(2-ethylhexyl) phthalate | C24 H38 O4 | 390 | 8.85 | 1.68 | 0.56 | 0.046 |
| Cyclopentenone | C5 H6 O | 82 | 0.28 | 1.35 | 0.52 | 0.043 |
| (-)-Isopiperitenone | C10 H14 O | 150 | 4.20 | 1.41 | 0.51 | 0.032 |
| 3-Vinyl-7-oxabicyclo[4.1.0]heptane | C8 H12 O | 124 | 5.93 | 1.32 | 0.50 | 0.035 |
| Adenine | C5 H5 N5 | 135 | 1.12 | 1.78 | 0.49 | 0.001 |
| N-Carbamoylputrescine | C5 H13 N3 O | 131 | 1.01 | 1.41 | 0.48 | 0.009 |
| (-)-Isopiperitenone | C10 H14 O | 150 | 8.12 | 1.48 | 0.47 | 0.046 |
| saccharin | C7 H5 N O3 S | 183 | 4.81 | 1.35 | 0.47 | 0.041 |
| Beta-Alanine | C3 H7 N O2 | 89 | 0.93 | 3.13 | 0.47 | 0.024 |
| Spermidine | C7 H19 N3 | 145 | 0.91 | 1.68 | 0.45 | 0.012 |
| gamma-Aminobutyric Acid | C4 H9 N O2 | 103 | 0.92 | 1.45 | 0.44 | 0.019 |
| Thymine | C5 H6 N2 O2 | 126 | 1.73 | 1.39 | 0.44 | 0.001 |
| L-Glutamic acid | C5 H9 N O4 | 147 | 0.94 | 1.99 | 0.42 | 0.011 |
| NPYR | C4 H8 N2 O | 100 | 20.07 | 2.51 | 0.42 | 0.031 |
| 5-Valerolactone | C5 H8 O2 | 100 | 2.76 | 5.10 | 0.42 | 0.046 |
| Oleamide | C18 H35 N O | 281 | 13.12 | 10.28 | 0.42 | 0.031 |
| 2,5-Dimethyl-3-hexyne-2,5-diol | C8 H14 O2 | 142 | 6.93 | 2.03 | 0.39 | 0.038 |
| 2,5-Dimethyl-3-hexyne-2,5-diol | C8 H14 O2 | 142 | 5.92 | 1.86 | 0.38 | 0.036 |
| Cytidine;1-beta-delta-Ribofuranosyl-Cytosine | C9 H13 N3 O5 | 243 | 0.93 | 1.53 | 0.37 | 0.012 |
| OxidizedLatialuciferin | C13 H22 O | 194 | 8.35 | 1.91 | 0.35 | 0.048 |
| UNII:212JQJ15PS | C8 H12 | 108 | 13.66 | 1.32 | 0.34 | 0.024 |
| Sulcatone | C8 H14 O | 126 | 5.75 | 2.78 | 0.34 | 0.046 |
| OxidizedLatialuciferin | C13 H22 O | 194 | 5.58 | 1.94 | 0.34 | 0.036 |
| OxidizedLatialuciferin | C13 H22 O | 194 | 7.07 | 1.86 | 0.33 | 0.049 |
| 1-Phenylethylamine | C8 H11 N | 121 | 20.05 | 3.73 | 0.33 | 0.002 |
| OxidizedLatialuciferin | C13 H22 O | 194 | 5.42 | 1.63 | 0.30 | 0.047 |
| Sulcatone | C8 H14 O | 126 | 6.08 | 4.07 | 0.30 | 0.047 |
| Megastigma-4,6(E),8(Z)-triene | C13 H20 | 176 | 9.00 | 1.56 | 0.29 | 0.035 |
| OxidizedLatialuciferin | C13 H22 O | 194 | 6.77 | 1.92 | 0.29 | 0.047 |
| Sulcatone | C8 H14 O | 126 | 8.39 | 1.73 | 0.25 | 0.029 |
| 4-Nonanoylmorpholine | C13 H25 N O2 | 227 | 13.96 | 2.17 | 0.21 | 0.046 |
| 2,6-di-tert-butyl-4-ethylphenol | C16 H26 O | 234 | 1.53 | 1.31 | 0.20 | 0.042 |
| Sulcatone | C8 H14 O | 126 | 0.66 | 3.14 | 0.19 | 0.036 |
| N-Undecanoylglycine | C13 H25 N O3 | 243 | 13.97 | 2.03 | 0.17 | 0.034 |
| 1,2,2,6,6-Pentamethyl-4-piperidinyl acrylate | C13 H23 N O2 | 225 | 13.73 | 2.68 | 0.16 | 0.038 |
| Sulcatone | C8 H14 O | 126 | 2.58 | 4.25 | 0.15 | 0.027 |
| Dicetylamine | C32 H67 N | 466 | 13.64 | 1.74 | 0.14 | 0.030 |
| PALMITYLSTEARYLAMINE | C34 H71 N | 494 | 14.34 | 2.63 | 0.08 | 0.030 |
| 4-Nonanoylmorpholine | C13 H25 N O2 | 227 | 13.74 | 2.93 | 0.07 | 0.033 |
| n2-octadecyl-1,3,5-triazine-2,4,6-triamine | C21 H42 N6 | 378 | 15.98 | 2.18 | 0.05 | 0.030 |

Calc. MW, calculate the molecular weight; RT [min], retention time; VIP, Value importance in Projection; FC S1_6/S3_4, fold change when group S1_6 compared with group S3_4.


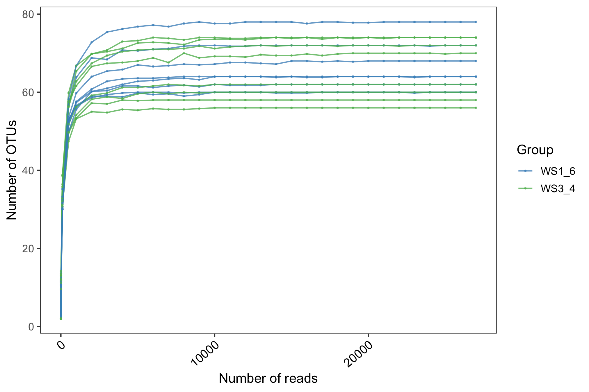


**Supplementary Figure 1.**. Rarefaction curves of methanogenic bacterial communities of low (WS1_6) and high (WS3_4) energy group yak.
